# Association of C-reactive protein to albumin ratio with all-cause and cardiovascular mortality in patients with chronic kidney disease stages 3–5

**DOI:** 10.1265/ehpm.24-00329

**Published:** 2025-03-20

**Authors:** Jie Liu, Jin Zhao, Jinguo Yuan, Zixian Yu, Yunlong Qin, Yan Xing, Qiao Zheng, Yueru Zhao, Xiaoxuan Ning, Shiren Sun

**Affiliations:** 1 Department of Nephrology, Xijing Hospital, The Fourth Military Medical University, Xi’an, Shaanxi, China; 2 Medical School, Yan’an University, Yan’an, Shaanxi, China; 3 Medicine School of Xi’an Jiaotong University, Xi’an, Shaanxi, China; 4 Department of Geriatrics, Xijing Hospital, Fourth Military Medical University, No. 127 Chang le West Road, Xi’an, 710032, Shaanxi, China

**Keywords:** Chronic kidney disease, NHANES, C-reactive protein to albumin ratio, Cardiovascular disease, Mortality

## Abstract

**Background:**

Chronic kidney disease (CKD) poses a major global health challenge, often foreshadowing poor patient outcomes. The C-reactive protein to albumin ratio (CAR) serves as a pivotal biomarker, demonstrating a strong correlation with adverse outcomes in cardiovascular disease (CVD). This study sought to examine the correlation between CAR and the risk of all-cause and cardiovascular mortality in patients with CKD stages 3–5.

**Methods:**

This study utilized data of CKD patients from the National Health and Nutrition Examination Survey (NHANES) from 1999 to 2010, with follow-up to December 31, 2019. The optimal CAR cutoff value was identified utilizing the method of maximally selected rank statistics. Multivariable Cox proportional hazards regression model, restricted cubic splines (RCS) model, and subgroup analysis were employed to assess the association between CAR and mortality among CKD patients.

**Results:**

During a median (with interquartile range) follow-up period of 115 (112,117) months among 2,841 CKD individuals, 1,893 deaths were observed, including 692 deaths due to CVD events. Based on the RCS analysis, a non-linear correlation was observed between CAR and mortality. Using 0.3 as the optimal CAR cutoff value, the cohort was divided into high and low groups. In the fully adjusted model, CKD patients with high CAR values exhibited an elevated risk of all-cause mortality (hazard ratio [HR] 1.53, 95% confidence interval [CI] 1.28–1.83, *P* < 0.001) and cardiovascular mortality (HR 1.48, 95% CI 1.08–2.02, *P* = 0.014). Compared to the population aged >65 years (HR 1.32, 95% CI 0.99–1.76, *P* = 0.064), the risk of cardiovascular mortality was significantly higher in those aged ≤65 years (HR 2.19, 95% CI 1.18–4.09, *P* = 0.014) with elevated CAR levels.

**Conclusions:**

A notable correlation exists between the elevation of CAR and increased all-cause and cardiovascular mortality, suggesting its potential as an independent indicator for evaluating the prognosis of patients with CKD stages 3–5.

**Supplementary information:**

The online version contains supplementary material available at https://doi.org/10.1265/ehpm.24-00329.

## 1. Introduction

Chronic kidney disease (CKD) represents a major global health challenge, affecting approximately 8% to 16% of the worldwide population [1]. Over the period from 1990 to 2017, CKD ascended from being the 17th to the 12th most prevalent cause of death worldwide, resulting in an estimated annual death count of 1.2 million individuals [2]. CKD is characterized by a persistently estimated glomerular filtration rate (eGFR) below 60 mL/min/1.73 m^2^ for at least 3 months, typically corresponding to stages 3–5 of CKD, which account for approximately 45.1% of the CKD population [2, 3]. The progression of CKD extends beyond renal impairment but is closely linked to numerous risk factors for cardiovascular disease (CVD), such as high blood pressure, arteriosclerosis, and myocardial injury [4, 5]. Cardiovascular-related mortality is markedly elevated among late-stage CKD patients, accounting for approximately 50% of all causes of death, substantially increasing overall mortality rates [4, 5]. These circumstances serve as a reminder that further research and intensified efforts are still necessary for the prognostic monitoring of CKD.

Patients with CKD stages 3–5 occurred with relatively high mortality around the world, but the effective prognostic biomarkers are lacking in clinical practice. Serum C-reactive protein (CRP) and albumin concentration are two commonly used laboratory markers that respectively reflect systemic inflammation response and nutritional status [6, 7]. Elevated CRP levels are closely associated with bodily injury or infection and occur frequently in various diseases, linked to increased risk of cardiovascular mortality [8, 9]. Albumin, crucial for maintaining anti-inflammatory and antioxidant defenses, exhibits reduced concentration indicative of compromised protective functions, suggesting heightened risks of disease and mortality [10–12]. CRP and albumin reveal systemic inflammation and nutritional imbalance from different perspectives and are both age-related, influencing the pace of aging [13, 14]. Therefore, the composite index of CRP to albumin ratio (CAR), as a new comprehensive indicator reflecting systemic inflammation load, may hold predictive value for disease prognosis. Existing research shows that CAR is associated with adverse outcomes of various diseases such as brain injury, Coronavirus Disease 2019 (COVID-19), and chronic obstructive pulmonary disease [15–17]. The role of CAR in CKD patients may hold significant clinical implications, but its specific role and potential value in predicting patient prognosis require further investigation.

The current studies investigate the relationship between CAR and CKD patients, with a particular focus on its predictive value for short-term acute manifestations [18, 19]. Given the increased mortality risks faced by individuals with CKD, there is a significant emphasis on long term efficacy assessment. Against this backdrop, in this study, we aim to explore CAR as a straightforward and effective biomarker in a large American sample with CKD, to predict the mortality risk among CKD stages 3–5 patients, while establishing a novel marker closely linked to the long-term prognosis of CKD.

## 2. Methods

### 2.1. Database and participants

This cross-sectional research employed publicly accessible data garnered from the National Health and Nutrition Examination Survey (NHANES) within the United States. The NHANES, conducted by the Centers for Disease Control and Prevention (CDC), is a multi-stage, stratified, and complex selection process designed to collect health, nutritional, and pertinent data from the national population. The investigation compiled data from the NHANES cohort across six successive cycles (1999–2000, 2001–2002, 2003–2004, 2005–2006, 2007–2008, and 2009–2010). The ethics review board of the National Centre for Health Statistics (NCHS) approved the NHANES protocol, with all individuals consenting in writing before engaging in the study.

### 2.2. Definition of CKD and CAR

For the diagnosis of CKD stages 3–5, the Chronic Kidney Disease Epidemiology Collaboration (CKD-EPI) equation is used for determining eGFR, with a threshold of less than 60 mL/min/1.73 m^2^ being the definitive criterion [20]. The CRP levels were determined using the Behring Nephelometer, with CRP quantified through latex-enhanced nephelometry [21]. As for the measurement of serum albumin concentration, there were variations in the reagent systems and interference factor treatments between 1999–2002 and 2003–2010. Both methods were based on the formation of complexes between albumin and Bromcresol Purple (BCP), with albumin concentration estimated by monitoring changes in absorbance. Compute the ratio of CRP (mg/dl) to albumin (g/dl) to obtain CAR (mg/g). There is no established cutoff value to define high and low CAR values. We used the Maximum Selected Rank Statistics method (MSRSM) to calculate the optimal cut-off value of CAR. This statistical approach centers on the Log-rank test and represents the strongest association with survival rates [22, 23]. The optimal cut-off value of CAR was used to divide participants into two distinct categories.

### 2.3. Covariates

Demographic, lifestyle, and comorbidity data of participants were carefully collected and analyzed as various covariates that could potentially influence the outcomes. Additionally, height and weight measurements were obtained from participants during the assessment period. As part of NHANES data, serum samples were carefully collected, providing laboratory data, and rigorous protocols were maintained during blood collection and analyses. Diabetes is defined as being diagnosed by a doctor, or having a glycated hemoglobin A1c (HbA1c) level ≥6.5%, or a fasting plasma glucose concentration ≥126 mg/dL [24]. Hypertension is defined as either taking antihypertensive drugs at baseline or having a systolic blood pressure ≥130 mmHg and/or a diastolic blood pressure ≥80 mmHg [25]. Dyslipidemia is defined as triglyceride levels of ≥150 mg/dL, high-density lipoprotein cholesterol levels of <40 mg/dL, low-density cholesterol levels of ≥130 mg/dL, or cholesterol levels of ≥200 mg/dL [15]. The particulars of pertinent definitions, such as body mass index (BMI) and poverty income ratio (PIR), are delineated in the Supplementary Method (Supplementary Method).

### 2.4. Ascertainment of outcomes

We accessed and obtained the mortality datasets from NHANES for public use up to December 31, 2019, and cross-referenced by NCHS with the National Death Index (NDI) death certificate records. Specific-cause mortality was ascertained utilizing the 10th Revision of the International Classification of Diseases Statistics (ICD-10), with cardiovascular mortality encompassing deaths attributed to heart diseases (codes I00–I09, I11, I13, I20–I51) and stroke-related pathologies (codes I60–I69) [26].

### 2.5. Statistical analysis

To address the complex sampling strategy employed in NHANES, specific sample weights (1/6* WTMEC2YR) were utilized, compensating for unequal probabilities of selection and varying rates of non-response. We categorized continuous variables, except age, eGFR, CRP, and albumin, with missing categorical data handled as a distinct category within their respective variable types. For continuous data, statistical description used the median and interquartile range (IQR) and for categorical variables, proportions were computed separately for each group. Wilcoxon tests and chi-square tests were used to evaluate differences among groups.

Using weighted multivariable Cox proportional hazards regression models for survival analyses, the purpose was to measure the influence of CAR on individual mortality risk and quantify this impact by calculating the hazard ratio (HR) and 95% confidence interval (CI). Building on fully adjusted model, subgroup analyses and interaction analyses were conducted to evaluate the mortality risk and potential interactions in different populations. Weighted Cox regression analyses were performed on the correlation between CAR and mortality risk in 12 subgroups, including age, gender, race, alcohol intake, smoking status, BMI, PIR, education level, presence of diabetes, hypertension, dyslipidemia, and eGFR levels. Sensitivity analyses conducted by excluding participants who might affect the reliability of the model results (those who died within two years, eGFR <15 mL/min/1.73 m^2^) revealed the extent of reliance of model outcomes on these subsets of individuals.

Use Kaplan-Meier curves and log-rank analyses to compare the prognosis between high and low CAR value cohorts, to examine the correlation between CAR levels and clinical prognosis, and to test the significance of this relationship. In addition, we applied restricted cubic spline (RCS) fitted for adjusted Cox proportional hazards models with four knots to detect potential associations between the CAR and all-cause and CVD mortality, using the R package “plotRCS” for this analysis. These analyses utilized R (version 4.3.0), with significance level set at *P* < 0.05.

## 3. Results

### 3.1. Baseline characteristics of the study population

**Fig. 1:**
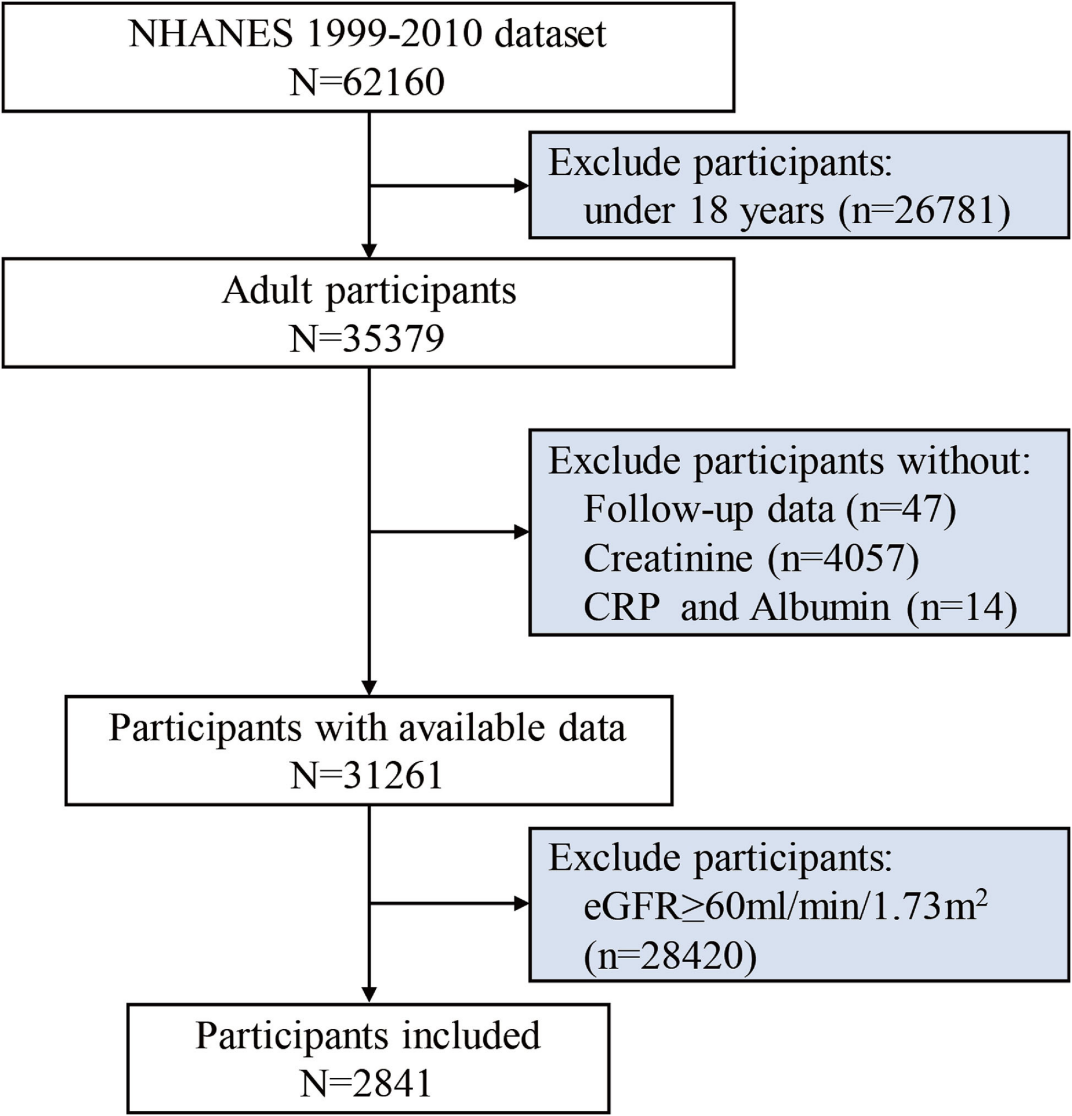
Participant selection process flowchart. Abbreviation: NHANES, National Health and Nutritional Examination Survey; CAR, C-reactive protein to albumin ratio; eGFR, estimated glomerular filtration rate.

In our analysis, 62,160 individuals participated, including 31,261 participants aged 18 years or older with complete follow-up data, serum creatinine, CRP, and albumin levels. Excluded were participants who had an eGFR ≥60 mL/min/1.73 m^2^. In total, 2841 participants with available data who had CKD stages 3–5 were involved in the analysis (Fig. 1).

From the ultimately analyzed study cohort, we divided it into two groups based on the optimal cutoff value of 0.30: a subgroup with CAR >0.30, termed the high-value group (n = 370), and another subgroup with CAR ≤0.30, termed the low-value group (n = 2,471). Over a median (with IQR) follow-up period of 115 (112,117) months, there were 1,893 deaths (weighted 60.7%), including 692 deaths (weighted 22.2%) from CVD events.

In the study of CKD population, the majority of patients have hypertension (83.0%) and dyslipidemia (65.7%), with diabetes also affecting 29.0% of them. Among patients in the CAR >0.30 group, there is an increased representation of Black patients, poorer economic status, higher prevalence of smoking and obesity, lower eGFR, higher CRP levels and lower albumin levels, and a higher proportion of diabetes patients. The remaining baseline characteristics were presented in Table 1.

**Table 1:** Baseline characteristics of participants with CKD stages 3–5 in the NHANES 1999–2010 cohort.

**Characteristic**	**Overall**	**CAR levels (mg/g)**		**P value**

		**Low-value group (≤0.30)**	**High-value group (>0.30)**	
**Participants, n**	2841	2471	370	
**CAR, mg/g**	0.15 (0.14, 0.16)	0.08 (0.07, 0.08)	0.68 (0.61, 0.75)	<0.001
**eGFR, mL/min/1.73 m^2^**	46.7 (46.2, 47.3)	47.4 (46.9, 47.9)	41.8 (39.9, 43.6)	<0.001
**Age, years**	71.1 (70.5, 71.7)	71.2 (70.6, 71.9)	70.1 (68.6, 71.5)	0.133
**Sex, n(%)**				0.279
Male	1360 (41.0)	1199 (41.3)	161 (37.6)	
Female	1481 (59.0)	1272 (58.7)	209 (62.4)	
**Race, n(%)**				<0.001
Mexican American	334 (4.6)	285 (4.7)	49 (4.7)	
Other Hispanic	68 (3.1)	63 (3.3)	5 (1.3)	
Non-Hispanic White	1706 (77.1)	1518 (78.0)	188 (69.9)	
Non-Hispanic Black	733 (15.2)	605 (14.0)	128 (24.1)	
**Smoking status, n(%)**				<0.001
Nonsmoker	1396 (51.2)	1248 (52.9)	148 (38.7)	
Former smoker	1150 (38.6)	989 (38.0)	161 (43.2)	
Current smoker	292 (10.1)	232 (9.1)	60 (18.1)	
Missing	3 (0.1)	2 (0.0)	1 (0.1)	
**Alcohol intake, n(%)**				0.013
None	557 (20.0)	488 (20.1)	69 (19.1)	
Moderate	772 (29.6)	690 (30.3)	82 (24.9)	
Heavy	214 (8.5)	189 (8.6)	25 (7.3)	
Binge	53 (2.1)	49 (2.3)	4 (0.8)	
Missing	1245 (39.8)	1055 (38.8)	190 (47.9)	
**Education level, n(%)**				0.001
Less than high school	727 (19.1)	609 (17.9)	118 (27.4)	
High school	521 (18.2)	457 (18.5)	64 (16.3)	
College or higher	1584 (62.5)	1398 (63.3)	186 (55.8)	
Missing	9 (0.2)	7 (0.1)	2 (0.4)	
**BMI, kg/m^2^, n(%)**				<0.001
<25.0	709 (24.9)	623 (25.2)	86 (22.2)	
25.0–30.0	1002 (35.9)	921 (37.8)	81 (21.5)	
>30.0	984 (35.3)	815 (33.4)	169 (48.9)	
Missing	146 (3.9)	112 (3.5)	34 (7.4)	
**PIR, n(%)**				0.016
<1.30	763 (21.1)	635 (20.3)	128 (26.9)	
1.31–3.50	1176 (42.0)	1027 (41.8)	149 (43.5)	
>3.50	648 (28.6)	587 (29.6)	61 (21.1)	
Missing	254 (8.3)	222 (8.2)	32 (8.5)	
**CRP, mg/dl**	0.6 (0.54, 0.62)	0.3 (0.30, 0.33)	2.5 (2.29, 2.79)	<0.001
**Albumin, g/dl**	4.1 (4.10, 4.13)	4.2 (4.13, 4.17)	3.8 (3.79, 3.88)	<0.001
**Diabetes, n(%)**	940 (29.0)	794 (27.5)	146 (40.0)	<0.001
**Hypertension, n(%)**	2111 (83.0)	2094 (82.3)	317 (88.1)	0.075
**Dyslipidemia, n(%)**	1823 (65.7)	1574 (65.1)	249 (70.4)	0.203

Values are median (interquartile range, IQR) for continuous variables or numbers (weighted %) for categorical variables. Abbreviation: NHANES, National Health and Nutritional Examination Survey; CVD, cardiovascular disease; CKD, chronic kidney disease. BMI, body mass index; PIR, poverty income ratio; eGFR, estimated glomerular filtration rate; CRP, C-reactive protein; CAR, C-reactive protein to albumin ratio.

### 3.2. Cutoff values

Across the full study cohort, a cutoff value of 0.30 (for all-cause mortality) and 0.34 (for CVD mortality) was calculated (Figs. S1A and S1B). Further stratification based on all-cause mortality revealed consistent cutoff values for females at 0.30 (Fig. S1C) and a slight variation for males at 0.26 (Fig. S1D). When stratified by age, the cutoff value for individuals aged ≤65 years was 0.27 (Fig. S1E) and for those aged >65 years, it was lower at 0.17 (Fig. S1F). Therefore, considering the main demographic characteristics, a cutoff value of 0.30 was further determined for grouping.

### 3.3. Mortality in relation to CAR

In the unadjusted continuous model (Model 1), the analysis of CAR showed that with each unit increase in CAR, there was a significant increase in the risk of all-cause mortality (HR 1.48, 95% CI 1.24–1.76, *P* < 0.001) and cardiovascular mortality (HR 1.56, 95% CI 1.29–1.89, *P* < 0.001). In Model 4 adjusting for basic demographic information, smoking and drinking behavior, BMI, economic status, education level, diabetes, hypertension, and dyslipidemia, an elevation of one unit in CAR was linked to a 43% increase in all-cause mortality (HR 1.43, 95% CI 1.20–1.70, *P* < 0.001) and a 55% increase in cardiovascular mortality (HR 1.55, 95% CI 1.28–1.87, *P* < 0.001) (Table 2). The categorical model indicates that, after adjusting for basic demographic information (Model 2), the high-value group had a significantly increased risk of all-cause mortality (HR 1.70, 95% CI 1.40–2.05, *P* < 0.001) and cardiovascular mortality (HR 1.66, 95% CI 1.21–2.27, *P* = 0.002) compared to the low-value group (HR 1.00). Similarly, in Model 4, the HRs and 95% CIs for all-cause mortality and cardiovascular mortality in the high-value group were 1.53 (1.28, 1.83) and 1.48 (1.08, 2.02), respectively, with *P* values <0.001 and equal to 0.014 (Table 2).

**Table 2:** Associations of CAR with all-cause and cardiovascular mortality in patients with CKD stages 3–5.

	**No. of Events**	**HR (95% CI) *P* value**			

		**Model 1**	**Model 2**	**Model 3**	**Model 4**
**All-cause mortality**					
CAR (continuous)	1893	1.48 (1.24, 1.76) <0.001	1.49 (1.23, 1.81) <0.001	1.42 (1.19, 1.69) <0.001	1.43 (1.20, 1.70) <0.001
CAR (categorical)					
High-value	279	1.60 (1.34, 1.91) <0.001	1.70 (1.40, 2.05) <0.001	1.54 (1.29, 1.85) <0.001	1.53 (1.28, 1.83) <0.001
**Cardiovascular mortality**					
CAR (continuous)	692	1.56 (1.29, 1.89) <0.001	1.57 (1.28, 1.91) <0.001	1.54 (1.28, 1.86) <0.001	1.55 (1.28, 1.87) <0.001
CAR (categorical)					
High-value	106	1.57 (1.15, 2.15) 0.004	1.66 (1.21, 2.27) 0.002	1.49 (1.09, 2.04) 0.012	1.48 (1.08, 2.02) 0.014

Values are n or weighted HR (95% CI). Model 1 is unadjusted; Model 2 is adjusted for: Age, Sex and Race; Model 3 is adjusted for: Model 2 plus Alcohol intake, Smoking status, BMI, PIR, Education level; Model 4 is adjusted for: Model 3 plus Diabetes, Hypertension, and Dyslipidemia. Abbreviation: CAR, C-reactive protein to albumin ratio; CKD, chronic kidney disease; HR, hazard ratio; CI, confidence interval; BMI, body mass index; PIR, poverty income ratio.

RCS analysis further explored the association of CAR with mortality. It showed a non-linear relationship between CAR and all-cause mortality (*P* < 0.001), consistent across two age groups (≤65 years: *P* = 0.006; >65 years: *P* < 0.001) (Fig. S2). Regarding the association of CAR with cardiovascular mortality, a non-linear relationship was observed between CAR and mortality in the entire population and ≤65-year-old population, with *P* value being 0.018 and 0.030, respectively. In the >65-year-old population, CAR exhibited a linear relationship with cardiovascular mortality (*P* = 0.118) (Fig. S2).

### 3.4. Survival analysis of CAR and mortality

**Fig. 2:**
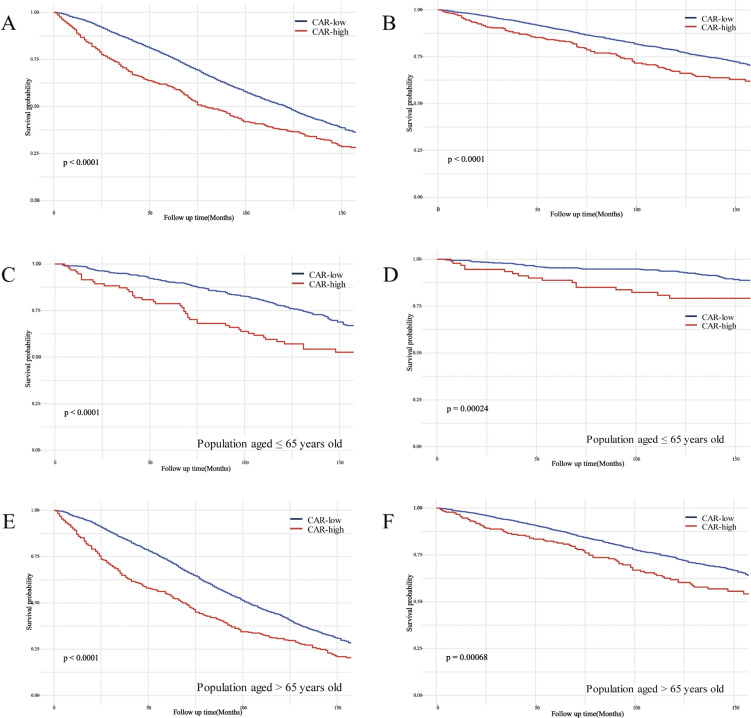
Kaplan-Meier curves of the survival rate between low and high CAR groups. (A) all-cause mortality in all participants; (B) CVD mortality in all participants; (C) all-cause mortality in the population aged ≤65; (D) CVD mortality in the population aged ≤65; (E) all-cause mortality in the population aged >65; (F) CVD mortality in the population aged >65. CAR, C-reactive protein to albumin ratio.

Based on the Kaplan-Meier curves, individuals with low CAR values among the CKD population showed significantly higher overall survival and cardiovascular disease survival probabilities compared to individuals with high CAR values, both with *P* < 0.0001 (Fig. 2A and B). Additionally, significant differences in survival probabilities were observed in both age strata (≤65 years and >65 years) across the two CAR groups (Fig. 2C–F).

### 3.5. Subgroup analysis

**Fig. 3:**
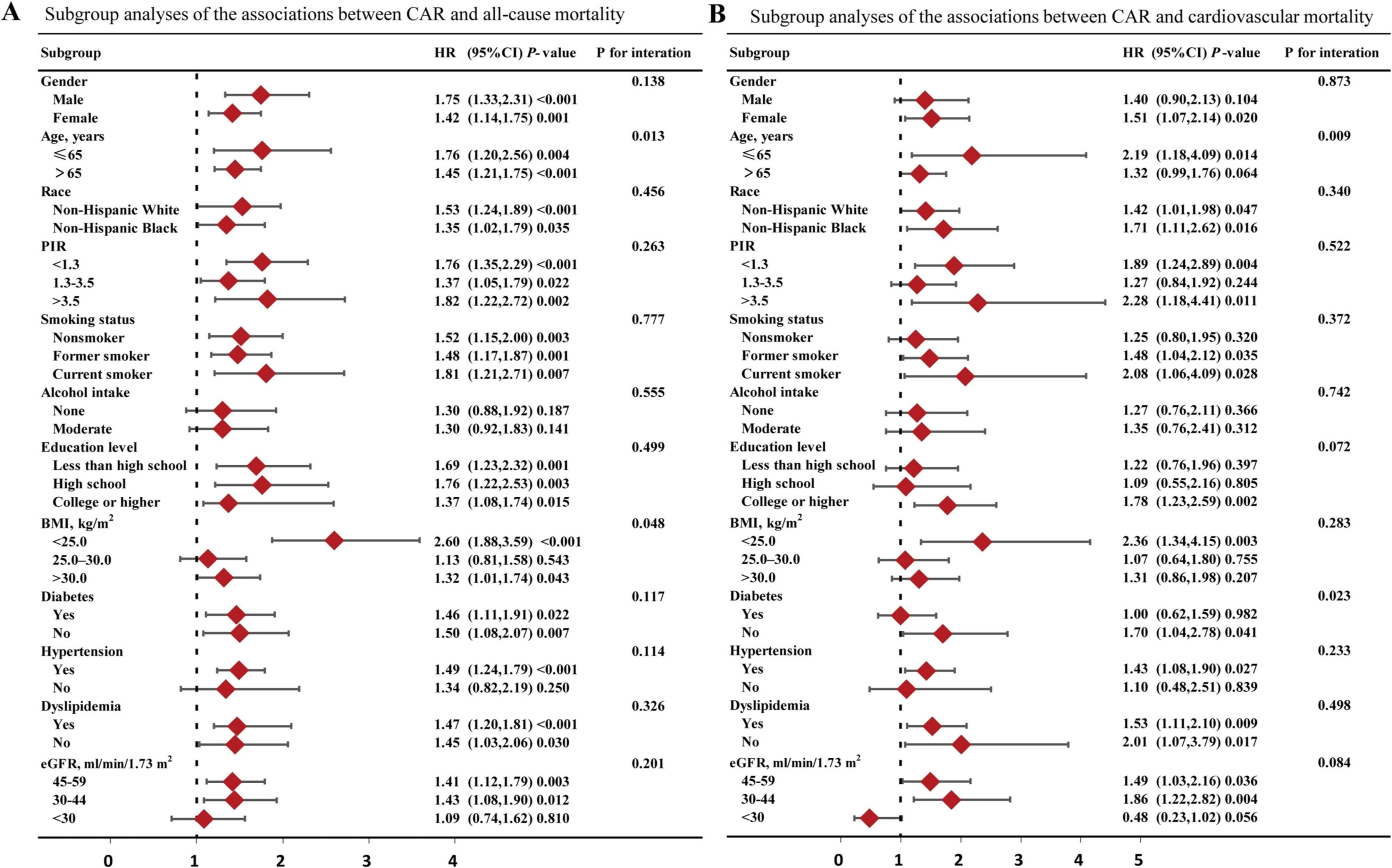
Subgroup analysis forest plot illustrating the association between high CAR group and mortality. (A) all-cause mortality; (B) CVD mortality. HRs were calculated using multivariable Cox regression models, adjusting for variables listed in the fully adjusted model (excluding stratified variables). Abbreviation: CAR, C-reactive protein to albumin ratio; CVD, cardiovascular disease; HR, hazard ratio; CI, confidence interval; PIR, poverty income ratio; BMI, body mass index; eGFR, estimated glomerular filtration rate.

As shown in Fig. 3, the correlation between CAR and the risk of mortality across various subgroups, generally aligned with our preliminary analysis, with the exception of a few subgroups where no statistical significance was observed. In terms of all-cause mortality, we found a significant interaction between CAR and age (*P* = 0.013) and BMI category (*P* = 0.048). The risk of mortality increased more significantly with elevated CAR in the younger group (≤65 years) within the age subgroups, with a HR of 1.76 (95%CI 1.20–2.56, *P* = 0.004), compared to the older group (>65 years) with a HR of 1.45 (95%CI 1.21–1.75, *P* < 0.001). Within the BMI subgroups, a notable elevation in the risk of mortality with higher CAR levels was observed in individuals with a BMI below 25.0 (HR 2.60, 95% CI 1.88–3.59, *P* < 0.001), compared to those with a BMI of 25.0–30.0 (HR 1.13, 95% CI 0.81–1.58, *P* = 0.543) and those with a BMI >30.0 (HR 1.32, 95% CI 1.01–1.74, *P* = 0.043). Similarly, in the analysis of cardiovascular mortality, we also observed significant differences between age groups (*P* = 0.009). The younger group (HR 2.19, 95%CI 1.18–4.09, *P* = 0.014) had a much higher risk of cardiovascular mortality with increased CAR than the older group (HR 1.32, 95%CI 0.99–1.76, *P* = 0.064).

### 3.6. Sensitivity analysis

Results indicate that elevated CRP levels contribute to higher all-cause mortality (HR 1.10, 95% CI 1.05–1.15, *P* < 0.001), while albumin exhibits a protective effect (HR 0.43, 95% CI 0.36–0.50, *P* < 0.001). The conclusions regarding their correlation with cardiovascular mortality are also consistent (Table S1).

After excluding patients who died within two years (n = 2,576) and those exhibiting an eGFR falling below 15 mL/min/1.73 m^2^ (n = 2,753), the effect estimates from the analyses showed slight fluctuations, and the relationship between CAR and all-cause/cardiovascular mortality remained consistent (Tables S2 and S3).

## 4. Discussion

In our large-scale prospective study using the NHANES database, we demonstrated a significant correlation between elevated circulating levels of CAR among individuals with CKD stages 3–5 and an increased risk of all-cause and cardiovascular mortality after accounting for potential confounding factors. Further subgroup analysis revealed that compared to patients aged >65 years, individuals aged ≤65 years with higher CAR levels had an augmented risk of mortality. Therefore, this study underscores the critical role of CAR as a key predictive biomarker for adverse outcomes in CKD patients.

CAR has been found to be associated with atherosclerosis, myocardial ischemia, and coronary artery disease [27, 28]. In populations without pre-existing CVD, elevated CAR levels can predict the occurrence of CVD during long-term follow-up [29]. Among individuals not in the stable phase of CVD, increased CAR correlates positively with short-term (failure of vascular access reconstruction, and hemodynamic disturbances) and long-term (thrombosis formation) adverse events [30–33]. Additionally, Tanık VO et al. observed that an increase in baseline CAR is correlated with an approximately twofold higher risk of all-cause mortality in populations with heart failure [34]. In our study, we also identified a correlation between each unit increase in CAR and mortality, particularly noted a significant association with cardiovascular mortality risk.

In our research, we explored for the first time the association between the CAR levels and increased mortality risk in CKD patients, emphasizing the potential role of CAR in long-term prognosis. Existing research primarily focused on the incidence of CKD in the general population, as well as the relationship between the need for continuous renal replacement therapy and the increased mortality rate at different time intervals in the short term [35, 36]. These studies consistently confirm that elevated CAR levels may serve as an independent predictive tool, holding a pivotal position in assessing the overall survival status of CKD patients.

Elevated levels of CAR correlate with heightened risks of mortality, with potential biological mechanisms likely involving enhanced inflammatory responses, deteriorating nutritional status, and their interactions, all closely related to organ damage and infection issues [37, 38]. CRP, a nonspecific inflammatory biomarker synthesized by the liver, typically increases concomitantly with circulating inflammatory mediators such as interleukin-6 (IL-6), interleukin-1β (IL-1β), and tumor necrosis factor-α (TNF-α) [39]. By reducing nitric oxide generation within endothelial cells, increasing expression of endothelial cell adhesion molecules, and monocyte recruitment, CRP promotes the progression and instability of atherosclerosis and thrombosis, thereby increasing individual mortality risk [40, 41]. This finding is consistent with conclusions drawn from our study. Albumin, similarly synthesized by hepatic cells, participates in substance transport and metabolism, provides nutritional support, and acts as an antioxidant [42]. During trauma or inflammatory states, it regulates vascular permeability [43]. Decreased albumin levels typically indicate poor prognosis [44]. Therefore, elevated CAR may indicate an aggravated inflammatory burden, worsening cardiovascular damage progression, and increased mortality risk.

Age stratification analysis reveals a significant difference in cardiovascular mortality risk among CKD individuals stratified by the age of 65. For those ≤65 years old, the HR associated with high CAR is 2.19 (95% CI 1.18–4.09), whereas for those >65 years old, the HR is 1.32 (95% CI 0.99–1.76). The observed lack of significance in the latter group may be due to a smaller size, resulting in fluctuation, yet the overall trend remains consistent. The attenuation of this mortality risk may be linked to aging, physiological changes, and disease, which affect cardiovascular health to varying degrees. As individuals age, the impact of inflammation on CVD progression may weaken compared to other cumulative heart health risks. Furthermore, we also observed a linear correlation between CAR and cardiovascular mortality risk in those >65 years old. This indicates that the attenuated inflammatory effect in the elderly population has a more stable and sustained impact on cardiovascular risk. Monitoring CAR in clinical management allows for early detection of changes in inflammatory burden, personalized assessment of cardiovascular risk based on patient age, and implementation of interventions such as dietary modifications and inflammatory modulation therapy to reduce risk [45–47].

Comparison with existing studies reveals discrepancies in CAR thresholds associated with mortality rates. Sant’Ana M et al. observed in their study of 787 patients initiating hemodialysis that higher CAR (≥0.5) significantly correlates with increased 6-month mortality risk (HR 5.36, 95% CI 3.21–8.96) [37]. Our findings suggest that CAR >0.3, is linked to adverse survival outcomes. The differences in research findings indicate that CAR could be a pivotal biomarker for assessing mortality risk in CKD patients, with its predictive capability potentially influenced by disease progression and specific clinical conditions.

The strength of the NHANES database lies in its extensive sample size and comprehensive clinical data, enabling thorough data mining and in-depth analyses. Through the rigorous acquisition of CRP and albumin and the calculation of CAR, we have revealed a correlation between CAR, a novel biomarker, and mortality in CKD patients. Particularly noteworthy, we conducted multiple subgroup analyses to further elucidate the specific populations associated with CAR and mortality, obtaining consistent conclusions across the majority of the population.

In our study, we must also acknowledge the inherent limitations of observational research designs that prevent establishing a true causal relationship. Furthermore, CRP and albumin levels were measured only once, which can be influenced by various factors and may fluctuate over time, potentially underestimating the association between CAR and CKD mortality. Moreover, due to the lack of detailed information on chronic glomerulonephritis, autoimmune diseases, and hemodialysis in the database, we were unable to fully assess how these conditions might impact the relationship between CAR and mortality. Lastly, despite accounting for various potential confounders, residual confounding factors are difficult to completely eliminate. Future studies could further explore these limitations and seek solutions to refine our knowledge of the connection between CAR and the risk of mortality in CKD patients.

## 5. Conclusion

A significant correlation has been found linking elevated CAR levels to the mortality risk among CKD patients in stages 3–5, suggesting that monitoring and maintaining CAR levels within a lower range may be beneficial for the prognosis of CKD patients. In the future, it will be necessary to conduct clinical trials to validate the optimal range of CAR level for reducing the risk of all-cause and cardiovascular mortality.

## Abbreviations

CRP: C-reactive protein
CAR: C-reactive protein to albumin ratio
CKD: chronic kidney disease
CVD: cardiovascular disease
COVID-19: Coronavirus Disease 2019
NHANES: National Health and Nutritional Examination Survey
NDI: National Death Index
CDC: the Centers for Disease Control and Prevention
ICD-10: the International Classification of Disease Statistics, 10th Revision
BCP: Bromcresol Purple
IQR: interquartile range
HR: hazard ratio
CI: confidence interval
BMI: body mass index
PIR: poverty income ratio
RCS: restricted cubic spline
eGFR: estimated glomerular filtration rate
SII: systemic immune-inflammation index
NCHS: National Centre for Health Statistics
CKD-EPI: Chronic Kidney Disease Epidemiological Collaboration
IL-6: interleukin-6
IL-1β: interleukin-1β
TNF-α: tumor necrosis factor-α

## Supplementary information

**Additional file 1:** Supplementary Method.

**Additional file 2:** **Figure S1:** The CAR cutoff value calculated through maximum selection rank statistics. (A) based on all-cause mortality for all participants; (B) based on cardiovascular mortality for all participants; (C) based on all-cause mortality for females; (D) based on all-cause mortality for males; (E) based on all-cause mortality for the population aged ≤ 65; (F) based on all-cause mortality for the population aged > 65.

**Figure S2:** Restricted cubic splines for associations of CAR levels with all-cause and CVD mortality. (A) all-cause mortality in all participants; (B) CVD mortality in all participants; (C) all-cause mortality in the population aged ≤ 65; (D) CVD mortality in the population aged ≤ 65; (E) all-cause mortality in the population aged > 65; (F) CVD mortality in the population aged > 65. CAR, C-reactive protein to albumin ratio; HR, hazard ratio; CI, confidence interval.

**Additional file 3:** **Table S1.** Associations of CRP and Albumin with all-cause and cardiovascular mortality in patients with CKD stages 3–5.

**Additional file 4:** **Table S2.** Multivariable-adjusted analyses after excluding the patients who died within two years (n = 2,576).

**Additional file 5:** **Table S3.** Multivariable-adjusted analyses after excluding the patients with eGFR < 15 mL/min/1.73 m^2^ (n = 2,753).

## Declarations

### Availability of data and material

The data relevant to my research is stored in the publicly accessible repository on the NHANES website (https://wwwn.cdc.gov/nchs/nhanes/Default.aspx).
