# Supplementary material for: Association of C-reactive protein to albumin ratio with all-cause and cardiovascular mortality in patients with chronic kidney disease stages 3–5
Source: Environ Health Prev Med. 2025 Mar 20;30:21. doi: 10.1265/ehpm.24-00329 (PMC11955801; doi:10.1265/ehpm.24-00329)
Supplement: Supplementary file 1 — Additional file 1: Supplementary Method. [file ehpm-30-021-s001.docx]

**Supplementary Method**

The smoking status is classified into three categories based on individual self-reported data on whether they have smoked at least 100 cigarettes in their lifetime and their current smoking behavior: never smokers, former smokers, and current smokers.

The alcohol intake was categorized into four groups based on the drinking habits of people in the self-reported data: no alcohol consumption, moderate alcohol consumption (1 drink per day for females or 1-2 drinks per day for males), heavy drinking (2 or more drinks per day for females or 3 or more drinks per day for males), or binge drinking (4 or more drinks per day for females or 5 or more drinks per day for males).

The body mass index (BMI) is calculated by dividing an individual’s weight (kg) by the square of their height (m^2^), and categorized into three groups based on the results: Normal weight ( < 25.0 kg/m²), Overweight (25.0-30.0 kg/m²), and Obese ( > 30.0 kg/m²).

The economic status of a family was assessed using the poverty income ratio (PIR), with higher PIR values indicating better economic conditions, which is categorized into three groups: < 1.30, 1.30-3.50, and > 3.50.

The alcohol intake was categorized into four groups based on the drinking habits of people in the self-reported data: no alcohol consumption, moderate alcohol consumption (1 drink per day for females or 1-2 drinks per day for males), heavy drinking (2 or more drinks per day for females or 3 or more drinks per day for males), or binge drinking (4 or more drinks per day for females or 5 or more drinks per day for males).
