# Supplementary figures and images for: Association of C-reactive protein to albumin ratio with all-cause and cardiovascular mortality in patients with chronic kidney disease stages 3–5
Source: Environ Health Prev Med. 2025 Mar 20;30:21. doi: 10.1265/ehpm.24-00329 (PMC11955801; doi:10.1265/ehpm.24-00329)

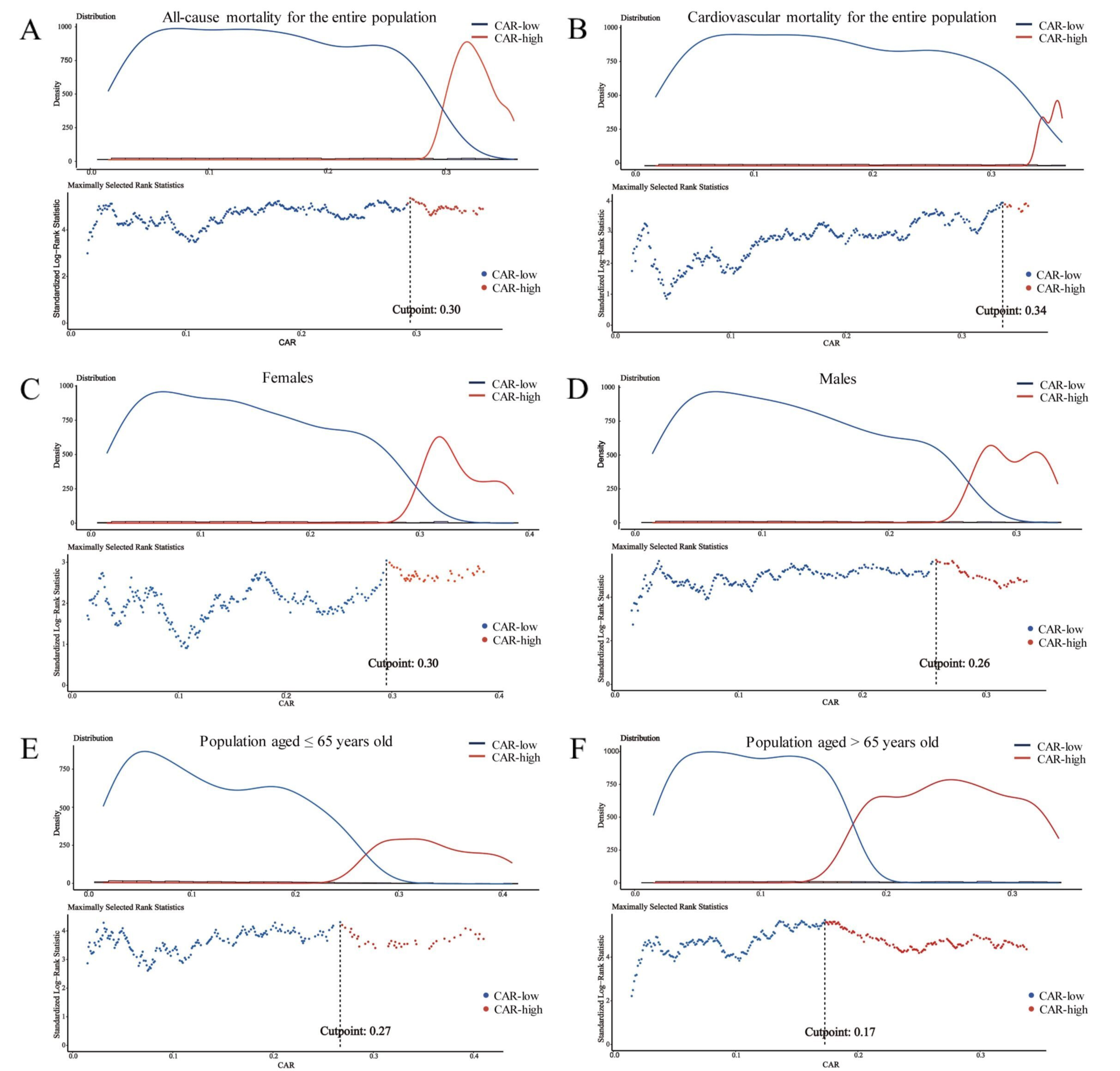

Supplement: Supplementary file 2 — Additional file 2: Figure S1: The CAR cutoff value calculated through maximum selection rank statistics. (A) based on all-cause mortality for all participants; (B) based on cardiovascular mortality for all participants; (C) based on all-cause mortality for females; (D) based on all-cause mortality for males; (E) based on all-cause mortality for the population aged ≤ 65; (F) based on all-cause mortality for the population aged > 65. [file ehpm-30-021-s002a.tif]

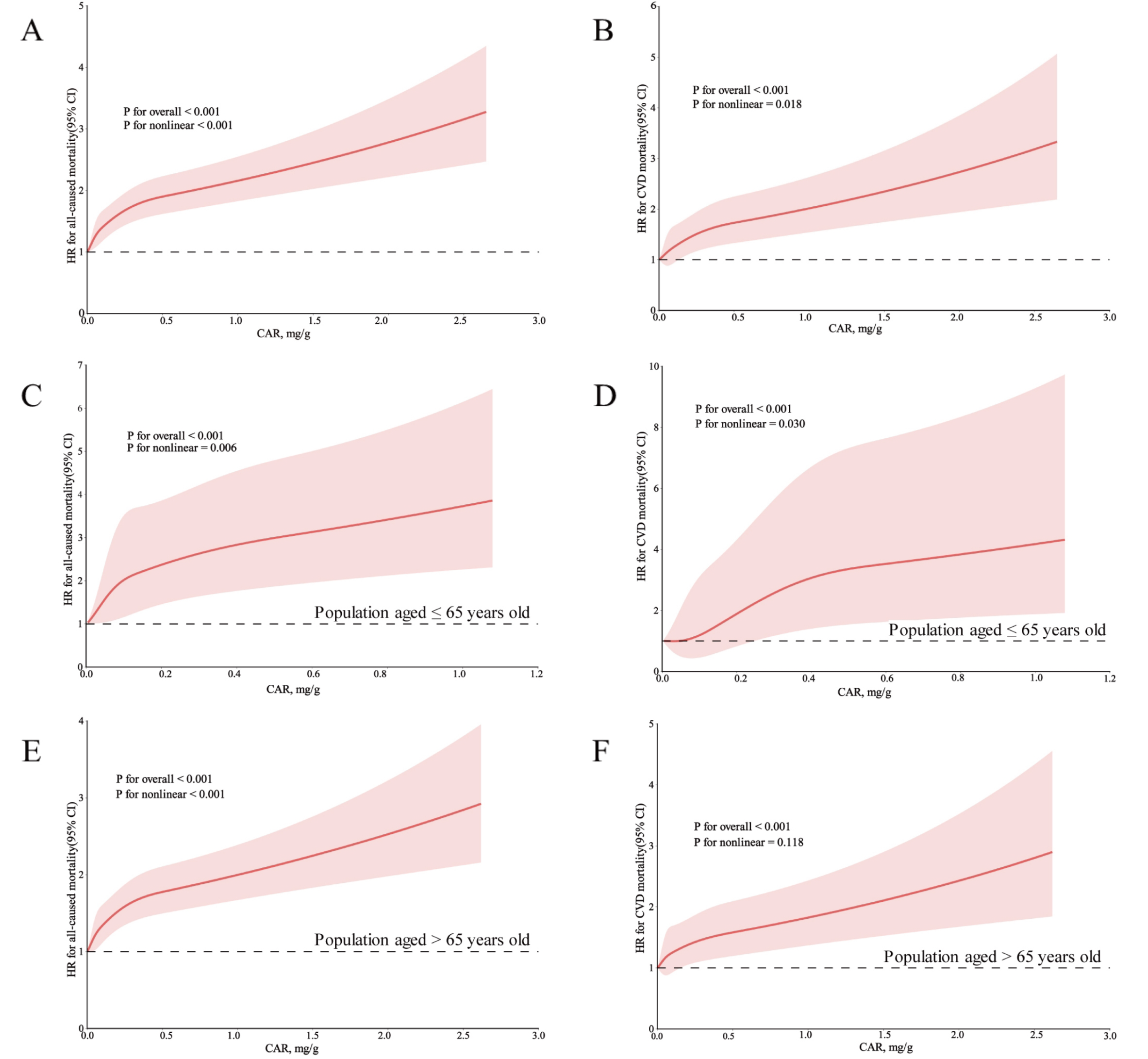

Supplement: Supplementary file 3 — Figure S2: Restricted cubic splines for associations of CAR levels with all-cause and CVD mortality. (A) all-cause mortality in all participants; (B) CVD mortality in all participants; (C) all-cause mortality in the population aged ≤ 65; (D) CVD mortality in the population aged ≤ 65; (E) all-cause mortality in the population aged > 65; (F) CVD mortality in the population aged > 65. CAR, C-reactive protein to albumin ratio; HR, hazard ratio; CI, confidence interval. [file ehpm-30-021-s002b.tif]
