# Supplementary material for: Association of C-reactive protein to albumin ratio with all-cause and cardiovascular mortality in patients with chronic kidney disease stages 3–5
Source: Environ Health Prev Med. 2025 Mar 20;30:21. doi: 10.1265/ehpm.24-00329 (PMC11955801; doi:10.1265/ehpm.24-00329)
Supplement: Supplementary file 4 — Additional file 3: Table S1. Associations of CRP and Albumin with all-cause and cardiovascular mortality in patients with CKD stages 3–5. [file ehpm-30-021-s003.docx]

**Table S1. Associations of CRP and Albumin with all-cause and cardiovascular mortality** **in patients with CKD stages 3-5.**

|  | **HR (95% CI) *P* value** | | | |
| --- | --- | --- | --- | --- |
|  | **Model 1** | **Model 2** | **Model 3** | **Model 4** |
| **All-cause mortality** |  |  |  |  |
| CRP | 1.11 (1.06, 1.16) <0.001 | 1.11 (1.06, 1.17) <0.001 | 1.10 (1.05, 1.15) <0.001 | 1.10 (1.05, 1.15) <0.001 |
| Albumin | 0.43 (0.35, 0.51) <0.001 | 0.37 (0.32, 0.44) <0.001 | 0.41 (0.35, 0.48) <0.001 | 0.43 (0.36, 0.50) <0.001 |
| **Cardiovascular mortality** |  |  |  |  |
| CRP | 1.13 (1.07, 1.19) <0.001 | 1.13 (1.07, 1.20) <0.001 | 1.13 (1.07, 1.19) <0.001 | 1.13 (1.07, 1.19) <0.001 |
| Albumin | 0.50 (0.37, 0.67) <0.001 | 0.45 (0.33, 0.60) <0.001 | 0.50 (0.37, 0.68) <0.001 | 0.51 (0.37, 0.70) <0.001 |
| Values are n or weighted HR (95% CI). Model 1 is unadjusted; Model 2 is adjusted for: Age, Sex and Race; Model 3 is adjusted for: Model 2 plus Alcohol intake, Smoking status, BMI, PIR, Education level; Model 4 is adjusted for: Model 3 plus Diabetes, Hypertension, and Dyslipidemia. Abbreviation: CRP, C-reactive protein; CKD, chronic kidney disease; HR, hazard ratio; CI, confidence interval; BMI, body mass index; PIR, poverty income ratio. | | | | |
