# Supplementary material for: Association of C-reactive protein to albumin ratio with all-cause and cardiovascular mortality in patients with chronic kidney disease stages 3–5
Source: Environ Health Prev Med. 2025 Mar 20;30:21. doi: 10.1265/ehpm.24-00329 (PMC11955801; doi:10.1265/ehpm.24-00329)
Supplement: Supplementary file 5 — Additional file 4: Table S2. Multivariable-adjusted analyses after excluding the patients who died within two years (n = 2,576). [file ehpm-30-021-s004.docx]

**Table S2. Multivariable-adjusted analyses** **after excluding the patients who died within two years (n = 2,576).**

|  |  | **HR (95% CI) *P* value** | | | |
| --- | --- | --- | --- | --- | --- |
|  | **No. of Events** | **Model 1** | **Model 2** | **Model 3** | **Model 4** |
| **All-cause mortality** |  |  |  |  |  |
| CAR (continuous) | 1628 | 1.35 (1.17, 1.55) <0.001 | 1.39 (1.18, 1.63) <0.001 | 1.31 (1.13, 1.52) <0.001 | 1.31 (1.14, 1.52) <0.001 |
| CAR (categorical) |  |  |  |  |  |
| High-value | 201 | 1.39 (1.16, 1.66) <0.001 | 1.48 (1.23, 1.78) <0.001 | 1.37 (1.14, 1.64) <0.001 | 1.34 (1.13, 1.61) <0.001 |
| **Cardiovascular mortality** |  |  |  |  |  |
| CAR (continuous) | 581 | 1.47 (1.24, 1.73) <0.001 | 1.49 (1.25, 1.78) <0.001 | 1.47 (1.24, 1.75) <0.001 | 1.47 (1.23, 1.76) <0.001 |
| CAR (categorical) |  |  |  |  |  |
| High-value | 75 | 1.36 (0.98, 1.89) 0.063 | 1.45 (1.05, 1.99) 0.023 | 1.34(0.98, 1.84) 0.069 | 1.33 (0.97, 1.82) 0.079 |
| Values are n or weighted HR (95% CI). Model 1 is unadjusted; Model 2 is adjusted for: Age, Sex and Race; Model 3 is adjusted for: Model 2 plus Alcohol intake, Smoking status, BMI, PIR, Education level; Model 4 is adjusted for: Model 3 plus Diabetes, Hypertension, and Dyslipidemia. Abbreviation: CAR, C-reactive protein to albumin ratio; HR, hazard ratio; CI, confidence interval; BMI, body mass index; PIR, poverty income ratio. | | | | | |
