# Supplementary material for: Association of C-reactive protein to albumin ratio with all-cause and cardiovascular mortality in patients with chronic kidney disease stages 3–5
Source: Environ Health Prev Med. 2025 Mar 20;30:21. doi: 10.1265/ehpm.24-00329 (PMC11955801; doi:10.1265/ehpm.24-00329)
Supplement: Supplementary file 6 — Additional file 5: Table S3. Multivariable-adjusted analyses after excluding the patients with eGFR < 15 mL/min/1.73 m2 (n = 2,753). [file ehpm-30-021-s005.docx]

**Table S3. Multivariable-adjusted analyses after excluding the patients with eGFR < 15 mL/min/1.73 m^2^ (n = 2,753).**

|  |  | **HR (95% CI) *P* value** | | | |
| --- | --- | --- | --- | --- | --- |
|  | **No. of Events** | **Model 1** | **Model 2** | **Model 3** | **Model 4** |
| **All-cause mortality** |  |  |  |  |  |
| CAR (continuous) | 1823 | 1.47 (1.23, 1.76) <0.001 | 1.48 (1.21, 1.79) <0.001 | 1.40 (1.17, 1.67) <0.001 | 1.40 (1.18, 1.68) <0.001 |
| CAR (categorical) |  |  |  |  |  |
| High-value | 256 | 1.54 (1.28, 1.84) <0.001 | 1.61 (1.33, 1.96) <0.001 | 1.47 (1.22, 1.78) <0.001 | 1.45 (1.21, 1.75) <0.001 |
| **Cardiovascular mortality** |  |  |  |  |  |
| CAR (continuous) | 669 | 1.56 (1.28, 1.89) <0.001 | 1.55 (1.27, 1.90) <0.001 | 1.54 (1.27, 1.87) <0.001 | 1.55 (1.27, 1.88) <0.001 |
| CAR (categorical) |  |  |  |  |  |
| High-value | 99 | 1.56 (1.14, 2.14) 0.006 | 1.62 (1.17, 2.24) 0.003 | 1.46(1.06, 2.03) 0.022 | 1.45 (1.04,2.00) 0.026 |
| Values are n or weighted HR (95% CI). Model 1 is unadjusted; Model 2 is adjusted for: Age, Sex and Race; Model 3 is adjusted for: Model 2 plus Alcohol intake, Smoking status, BMI, PIR, Education level; Model 4 is adjusted for: Model 3 plus Diabetes, Hypertension, and Dyslipidemia. Abbreviation: CAR, C-reactive protein to albumin ratio; HR, hazard ratio; CI, confidence interval; BMI, body mass index; PIR, poverty income ratio. | | | | | |
